# Calcitonin gene-related peptide: a possible biomarker in migraine patients with patent foramen ovale

**DOI:** 10.1186/s12883-024-03615-1

**Published:** 2024-04-16

**Authors:** Chaojie Li, Yu Yu, Ningning Li, Ya-Na Yin, Lianjun Zhang, Kehang Xie, Donghui Huang

**Affiliations:** 1 People’s Hospital of Xiangzhou District, Zhuhai, Guangdong province 519000 China; 2 Department of cardiothoracic surgery, Zhuhai Hospital of Integrated Traditional Chinese and Western Medicine, Zhuhai, Guangdong province 519000 China; 3 Department of Health Management Division, Zhuhai Maternal and Child Health Care Hospital, Zhuhai, Guangdong province 519000 China; 4 Institute of Integrated Chinese and Western Medicine, Zhuhai Hospital of Integrated Traditional Chinese and Western Medicine, Zhuhai, Guangdong province 519000 China; 5 Clinical Medical College of Acu-Moxi and Rehabilitation, Guangzhou University of Chinese Medicine, Guangzhou, Guangdong province 510080 China; 6 Department of Preventive Medicine, Zhuhai Hospital of Integrated Traditional Chinese and Western Medicine, Zhuhai, Guangdong province 519000 China; 7 Zhuhai Hospital of Integrated Traditional Chinese and Western Medicine, 208 Yuehua Road, Xiangzhou District, Zhuhai, Guangdong province 519000 China

**Keywords:** Migraine, Patent foramen ovale, Calcitonin gene-related peptide, Right to left shunt

## Abstract

**Background:**

Serum CGRP has been found to increase during migraine attack. However, whether CGRP can identify MA with PFO subtypes in MA remains unknown. This study aimed to investigate the differential expression of calcitonin gene-related peptide (CGRP) between migraine (MA) patients with and without patent foramen ovale (PFO), and to evaluate the predictive value of CGRP for MA with PFO.

**Methods:**

A total of 153 patients with MA, 51 patients with PFO and 102 patients without. Venous blood was drawn and HIT-6 score was calculated during the onset of MA, and blood routine, inflammatory indexes and serum CGRP were detected. The differences in serum markers and HIT-6 scores were compared between the two groups, and the risk factors of MA with PFO were determined by univariate and multivariate logistics regression. Furthermore, the correlation between CGRP level with right-to-left shunt (RLS) grades and headache impact test-6 (HIT-6) score in MA patients with PFO were assessed. Independent risk factors were screened out by multivariate Logistic regression analysis. We used the receiver operating characteristic (ROC) curve to analyze the diagnostic value of these risk factors in MA complicated with PFO.

**Results:**

The serum CGRP level and HIT-6 scores in the MA with PFO group were significantly higher than those in the MA group (*P* < 0.001). Multivariate regression analysis showed that CGRP was an independent risk factor for MA with PFO (OR = 1.698, 95% CI = 1.325–2.179, *P* < 0.001). CGRP values ​​increased with the increase of RLS grade(Spearmen rho = 0.703, *P* < 0.001). Furthermore, a positive correlation between CGRP and HIT-6 scores was found (Spearmen rho = 0.227; *P* = 0.016). ROC curve showed that the optimal cut-off value for diagnosing MA with PFO was 79 pg/mL, the area under the curve (AUC) for predicting MA with PFO was 0.845, with 72.55% sensitivity and 78.43% specificity.

**Conclusions:**

MA patients with PFO have higher serum CGRP level. elevated CGRP concentration was associated with higher RLS grade and increased HIT-6 score. Higher serum CGRP level has certain clinical value in predicting PFO in MA patients.

**Trial registration:**

This study was approved by the Ethics Committee of Zhuhai Hospital of Integrated Traditional Chinese and Western Medicine (Ethics batch number: 20,201,215,005).

## Introduction

The number of migraine (MA) patients worldwide has reached 1.1 billion in 2019, becoming the second most disabling disease [1]. Until now, MA treatment focuses on symptomatic pain relief, which is only effective in 30–50% of the MA population [2]. To be noted, it is reported that patients with MA with patent foramen ovale (PFO) can achieve a radical cure of headache by blocking PFO [3]. Given that the prevalence rate of MA with PFO varies between 14.6 and 66.5% in different studies, there is a possibility of missed diagnosis of MA with PFO [4]. Moreover, current methods [contrast transcranial Doppler(c-TCD), transthoracic echocardiography (TTE), and transesophageal echocardiography (TEE)] for diagnosing PFO depend on the physicians to recognize cardiac abnormality, and their diagnostic criteria are not unified [5]. Therefore, it is crucial to find biomarkers that can distinguish PFO subtypes from MA, in addition to improving the diagnostic level of cardiac color ultrasound physicians in PFO.

Calcitonin gene-related peptide (CGRP) is a bioactive peptide widely distributed in the central and peripheral nervous system. It can cause MA attacks by expanding blood vessels via the trigeminal pathway, controlling neuron excitability, sensitizing the peripheral and central nervous systems via degranulating mast cells, and enhancing pain perception [6, 7]. CGRP has been found to increase during migraine attack [8, 9]. It is suggested that CGRP could be used as a non-invasive marker for migraine [10]. Many vasoactive substances are usually excreted or metabolized through pulmonary circulation, but venous blood can be shunted into arterial blood through the PFO channel without circulation in the lungs. Some chemicals and hormones, such as 5-HT and CGRP, can bypass the pulmonary circulation and cross the blood-brain barrier directly, stimulating the trigeminal nerve and causing migraines [11, 12].Therefore, we speculate that the concentration of serum CGRP is higher in patients with MA. However, it is not clear whether CGRP, as the most potential biomarker of migraine, can identify MA with PFO subtypes in MA remains unknown.

This study aims to identify the correlation between plasma CGRP levels and MA with PFO attacks during the attack of MA, and to provide some evidence for the clinical identification of MA with PFO subtypes.

## Materials and methods

### Study setting and participants

**Fig. 1:**
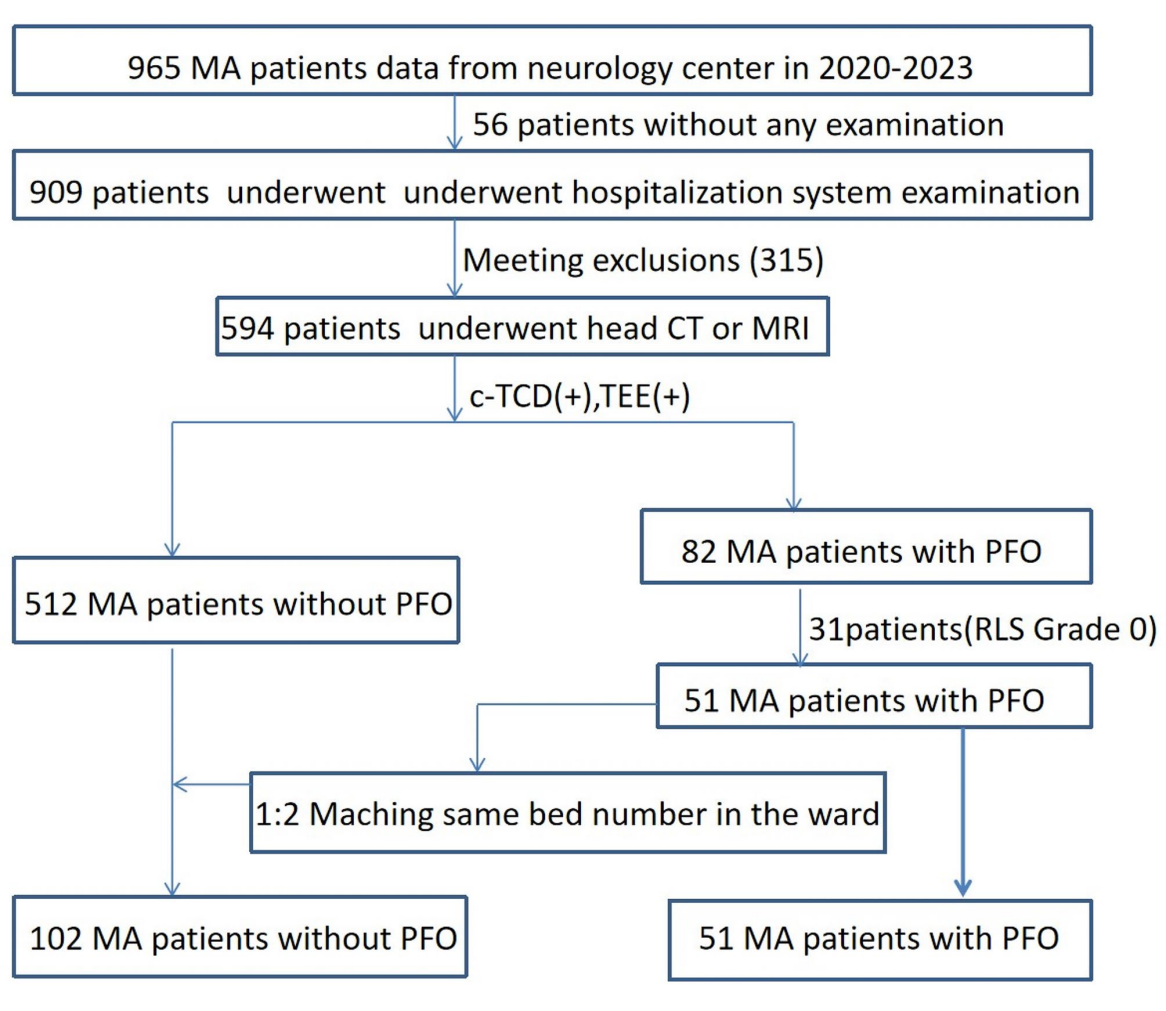
Flow chart for patient enrollment of the study

From January 2020 to January 2023, 51 patients diagnosed with MA and PFO by c-TCD foaming test combined with TEE examination in Zhuhai Hospital of Integrated Traditional Chinese and Western Medicine were selected. The data of MA patients with PFO and those without PFO with the same bed number were collected according to the 1:2 pairing principle(Fig. 1). All patients were systematically diagnosed by neurologists according to the guidelines [13]. This study as conducted in accordance with the guidelines of the World Medical Association Declaration of Helsinki (2000) and was approved by the Ethics Committee of Zhuhai Hospital of Integrated Traditional Chinese and Western Medicine (Ethics batch number: 20,201,215,005). All participants provided written informed consent.

### Inclusion and exclusion criteria

Inclusion criteria included: (1) the diagnostic criteria for migraine refer to the third edition of the International Headache Classification [14]; (2) positive c-TCD, TEE clearly diagnosed PFO and right-to-left shunt (RLS), the degree of shunt in TEE angiography is quantified in accordance with the microbubbles detected in the left atrium: Grade 0, no microbubbles are negative; Grade I, a small amount, 1–10 microbubbles; Grade II Moderate volume, 11–25 microbubbles; Grade III, large amount, more than 25 microbubbles or left atrium almost filled with microbubbles or left atrium opacity) [15]; (3) aged 18–80 years old; (4) able to cooperate with the completion of follow-up with complete and valid information; (5) electrocardiogram and other laboratory tests are normal.

Exclusion criteria included: (1) with headaches caused by other definite reasons; (2) pregnant or breastfeeding; (3) severe liver and kidney dysfunction, abnormal blood coagulation, autoimmune diseases, malignant tumors, or severe physical diseases, (4) cannot cooperate with the examination because of abnormal mental behavior or poor physical fitness; (4) incomplete follow-up data; (5) pulmonary arteriovenous fistula; (6) participate in other clinical research at the same time.

### Clinical information

General clinical data of the subjects were collected, including age, gender, basic medical history (essential hypertension, diabetes, coronary heart disease, atrial fibrillation), medication history, migraine aura, and smoking and drinking hobbies. All patients completed the head CT/MRI, ECG and foaming test. If the foaming test was positive, TEE was performed.

### Clinical and laboratory parameters

#### Headache scale score

Headache impact test-6 (HIT-6) [16]: Evaluate the negative evaluation of daily life caused by the onset headache of the patient, with a score ranging from 36 to 78 points. The score is positively correlated with the degree of disability impact, and a score above 60 means severe impact, 56–59 means significant impact, 50–55 means moderate impact, 36–49 means slight or no impact.

#### Collection and processing of blood samples

Fasting peripheral venous blood in the morning was drawn for detection.

General blood indicators: Complete blood cell counts were measured using the ethylenediaminetetraacetic acid blood sample method on a Toshiba analyzer (Hitachi High-Tech, Tokyo, Japan). Serum C-reactive protein (CRP), uric acid (UA) total cholesterol (TC), triglycerides (TG), high-density lipoprotein cholesterol (HDL) and low-density lipoprotein cholesterol (LDL) were measured with a Hitachi LST008 automatic biochemical analyzer. Fibrinogen (FIB) was determined using an automated coagulation analyzer (ACL-TOP-700, Wolfen, Spain).

CGRP collection and measurement: CGRP level was detected during the migraine attack period with EDTA anticoagulant tubes, followed by the centrifugation at a speed of 1000 r/min and at a temperature below 4 °C for 10 min. The separated supernatant was stored in a -80 °C refrigerator. Finally, radioimmunoassay was used to measure the concentration of plasma CGRP following the instructions of the kit (Human CGRP ELISA kit (ml026014), Shanghai Enzymatic Biology Co., Ltd.).

#### Statistical analysis

Data conforming to normal distribution was represented by mean ± standard deviation, and the t test was used for comparison between groups. Data not conforming to the normal distribution Otherwise, data was represented by median (Q25, Q75), and the Mann-Whitney U test was used for comparison between groups. The count data were expressed as cases (%), and the chi-square test or Fisher’s exact test was used for comparison between groups. Univariate and multivariable logistic regression models were used to assess the association between dependent variable (MA with POA (yes/no)) and variables. Correlation analysis was performed on CGRP and HIT-6 score as well as PFO diameter in MA patients with PFO. Pearson correlation analysis is carried out for the data in accordance with normal distribution, otherwise spearman correlation analysis is carried out. The area under the curve (AUC) of the receiver operator characteristic curve (ROC) was calculated and Youden Index was used to obtain the optimal cut-off value. *P* < 0.05 means significant difference. Statistical analysis was analyzed using GraphPad Prism 8 and SPSS 23.0.

## Results

### Comparison of general data and serum CGRP levels

A total of 153 patients were included in this study: 102 MA patients (MA group), and 51 MA patients with PFO (Table 1). There was no difference between the two groups in terms of demographic data, including gender, BMI, coronary heart disease, atrial fibrillation, hypertension, diabetes, and medication history (Table 1).

**Table 1:** The clinical characteristics of the study samples

Characteristics	MA with PFO(*n* = 51)	MA(*n* = 102)	P value
Age(years), mean(SD)	53.09(1.75)	47.79(1.17)	0.011
Male, n(%)	26(51)	49(48)	0.732
Alcohol consumption, n(%)	4(8)	13(13)	0.363
Current smoking, n(%)	15(29)	61(40)	0.192
Coronary artery disease, n(%)	3(6)	10(10)	0.546
Atrial fibrillation, n(%)	3(6)	8(8)	0.753
Hypertension, n(%)	13(26)	21(21)	0.492
Diabetes mellitus, n(%)	13(26)	22(22)	0.586
BMI, median (IQR)	23.26(21.16,26.26)	24.27(22.02,26.56)	0.392
Aura migraine, n(%)	21(41)	34(33)	0.341
Migraine years, median (IQR)	5(3,7)	5(3,7)	0.704
WBC(10^9^/Ul), median (IQR)	6.90(5.70,8.80)	6.70(5.50,8.20)	0.404
HGB (10 ^9^ /L), mean (SD)	142.51(13.10)	140.96(12.26)	0.517
RBC(10 ^9^ /L), median (IQR)	4.89(4.59,5.12)	4.64(4.35,4.97)	**0.011**
RDW (%),median (IQR)	12.77(12.30,13.75)	12.70(12.07,13.40)	0.178
PLT(10 ^9^ /L), mean (SD)	233.37(73.26)	225.20(53.14)	0.077
PDW, median (IQR)	12.90(11.30,15.70)	12.45(10.98,15.50)	0.383
MPV, median (IQR)	10.30(9.60,11.40)	10.00(9.40,10.53)	**0.023**
CRP (mg/L) median (IQR)	4.80(1.50,7.35)	2.35(0.90,4.20)	**0.005**
FIB(g/L), median (IQR)	2.70(2.23,2.98)	2.32(2.04,2.78)	**0.006**
UA(µmol/L), median (IQR)	342(272,387)	350(292,420)	0.318
TG(mmol/L), median (IQR)	1.16(0.86,1.93)	1.24(0.91,1.63)	0.745
TC(mmol/L), median (IQR)	4.59(3.73,5.17)	4.87(4.34,5.64)	**0.003**
LDL(mmol/L), median (IQR)	2.66(2.17,3.18)	2.74(2.37,3.14)	0.438
HDL(mmol/L), median (IQR)	1.26(1.04,1.47)	1.33(1.08,1.50)	0.406
CGRP(pg/mL), median (IQR)	83(78,88)	64(52,68)	**< 0.001**
HIT-6,median (IQR)	66(58,72)	60(52,68)	**0.002**
**Medication history**			
Antihypertensive therapy, n(%)	28(55)	53(52)	0.731
Antiglycemic therapy, n(%)	17(33)	36(35)	0.810
Anticoagulant therapy, n(%)	1(2)	3(3)	1.000
Oral painkillers, n(%)	46(90)	98(96)	0.161

BMI: body mass index, defined as weight in kilograms divided by the square of height in meters; WBC: white blood cells; HGB: haemoglobin; RBC: red blood cell; RDW: red blood cell distribution width; PLT: blood platelet; PDW : Platelet distribution width; MPV: Mean platelet volume; CRP: c reactive protrin; FIB: fibrinogen; UA: Uric acid; TG: triglycerid; TC: Tot Al Cholesterol; LDL: Low-Density Lipoprotein Cholesterol; HDL: High-Density LipoProtein Cholesterolol ; CGRP: calcitonin gene related peptide; HIT-6:headache impact test-6; The differences were considered significant if *P* value < 0.05

**Fig. 2:**
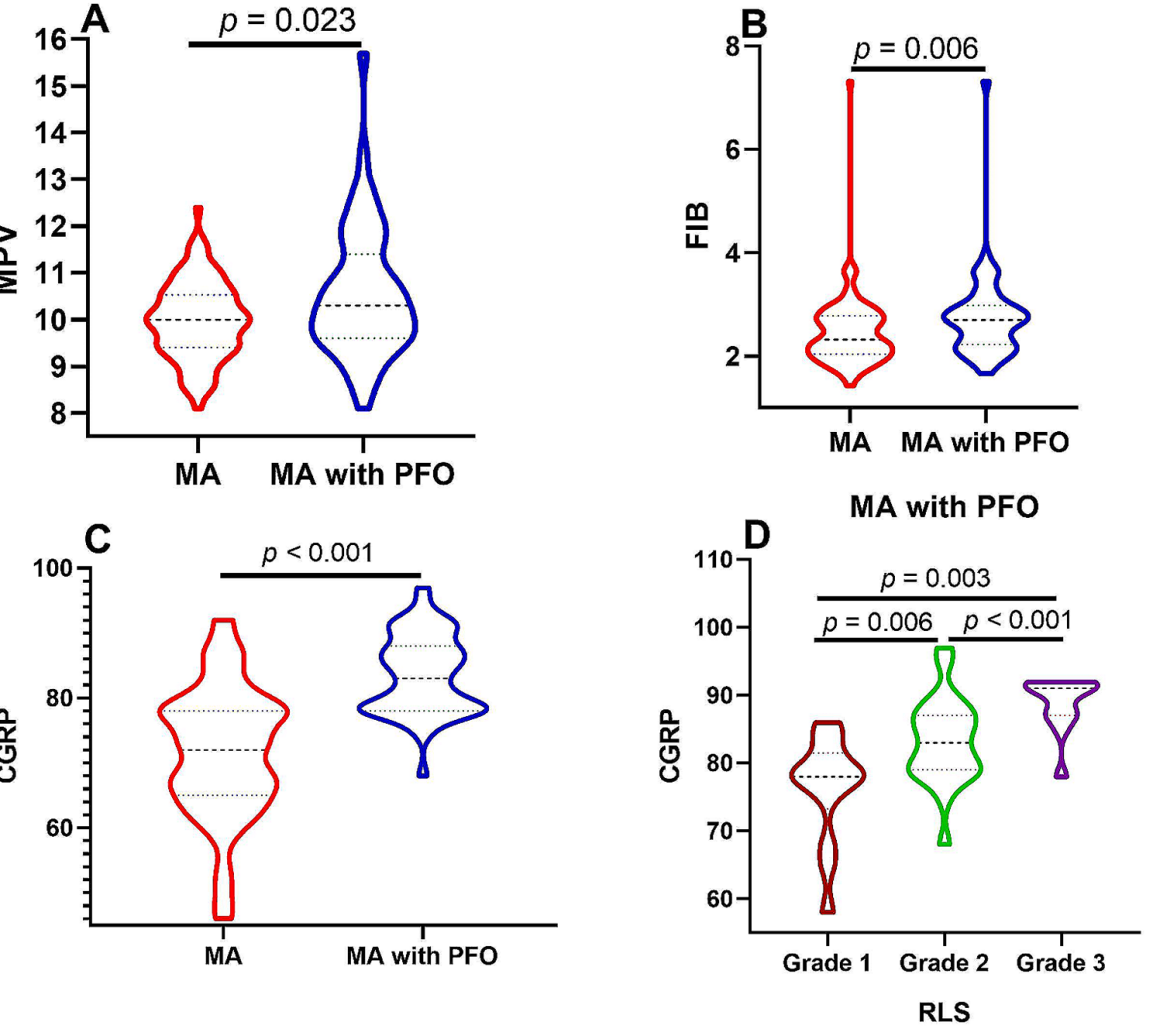
Comparison of MPV, FIB and CGRP between two groups and the relationship between CGRP and RLS in MA with PFO group

Serum CGRP levels in the MA with PFO group were significantly higher than those in the MA group (83(78,88) vs. 64(52,68), *P* < 0.001). Serum levels of RBC, MPV, CRP, FIB, and TC in the MA with PFO group were higher than those in the MA group (Table 1). In addition, the HIT-6 score of the MA with PFO group was higher than that of the MA group (*P* < 0.001) (Fig. 2 A-C).

### Risk factors for MA with PFO

Univariate logistic regression analysis showed that RBC, MPV, CRP, FIB, TC, CGRP and HIT-6 were associated with MA with PFO (Table 2, *P* < 0.05). After adjusting for significant variables in univariate logistic regression model, multivariate logistic regression analysis revealed that CGRP was an independent risk factor for MA with PFO (*P* < 0.001; OR = 1.698; 95%CI = 1.325–2.179).

**Table 2:** Risk factors for MA with PFO using Univariate and multivariate logistics regression analysis

Risk factors	Univariate logistics regression analysis			Multivariate logistics regression analysis		
	OR	95% CI	p Value	OR	95% CI	p Value
RBC	0.529	0.286 ~ 0.980	**0.043**	0.177	0.010 ~ 3.280	0.254
MPV	0.597	0.422 ~ 0.845	**0.004**	0.345	0.118 ~ 1.011	0.052
CRP	0.913	0.839 ~ 0.994	**0.035**	0.848	0.636 ~ 1.130	0.261
FIB	0.575	0.338 ~ 0.975	**0.040**	1.314	0.195 ~ 8.841	0.779
TC	1.908	1.276 ~ 2.854	**0.002**	1.643	0.352 ~ 7.672	0.528
CGRP	1.610	1.326 ~ 1.953	**< 0.001**	1.698	1.325 ~ 2.179	**< 0.001**
HIT-6	0.943	0.909 ~ 0.979	**0.002**	0.907	0.815 ~ 1.010	0.077

The differences were considered significant if *P* value < 0.05

### Relationship between CGRP value and RLS diversion level

In order to further explore the characteristics of CGRP in MA patients with PFO, we divided MA patients with PFO into 3 groups according to RLS classification (Grade 1, Grade 2, Grade 3, Table 3). Our results suggested that serum CGRP levels increased as the RLS grade increased (78 (73.25,81.5) vs. 83.25 (6.37) vs. 91 (87,92), all *P* < 0.05). (Fig. 2D)

**Table 3:** The clinical characteristics of the study samples

Characteristics	RLS			P^1^	P^2^	P^3^
	Grade 1 (*n* = 14)	Grade 2 (*n* = 24)	Grade 3 (*n* = 13)			
Age(years), mean(SD)	50.50(11.20)	55.13(14.08)	52.15(10.93)	0.313	0.953	0.339
Male, n(%)	7(50)	13(54)	6(0.46)	0.804	0.842	0.642
Alcohol consumption, n(%)	1(7.14)	2(8.33)	1(7.69)	0.896	0.957	0.210
Current smoking, n(%)	3(21.43)	9(37.50)	3(23.08)	0.304	0.918	0.371
CAD, n(%)	2(14.29)	0(0)	1(7.69)	0.057	0.586	0.168
AF, n(%)	2(14.29)	1(4.17)	0(0)	0.246	0.157	0.456
Hypertension, n(%)	3(21.43)	8(33.33)	2(15.38)	0.435	0.686	0.241
Diabetes mellitus, n(%)	3(21.43)	6(25)	4(30.77)	0.803	0.580	0.706
BMI, mean (SD)	23.81(3.37)	25.28(4.50)	22.35(2.52)	0.179	0.344	**0.029**
MA nMA, n(%)	5(35.17)	13(54.17)	3(23.08)	0.183	0.472	0.068
MA years, median (IQR)	5(2,8.25)	4(3,6.75)	6(4,7.5)	0.879	0.365	**0.017**
ASA, n(%)	1(7.14)	3(12.5)	1(7.69)	0.604	0.954	0.653
PFO diameter, median(IQR)	1.97(1.85,2.18)	2.20(2.07,2.38)	2.28(2.06,2.65)	**0.017**	**0.014**	**0.004**
PFO length, mean(SD)	12.22(2.45)	12.78(3.01)	12.87(2.50)	0.608	0.541	0.311
WBC(10^9^/Ul), median(IQR)	6.91(5.68,8.93)	6.80(5.34,9.00)	6.90(5.74,8.55)	0.928	0.364	0.975
HGB (10 ^9^ /L), mean (SD)	144.58(11.51)	144.00(12.30)	137.54(15.68)	0.822	0.533	0.111
RBC(10 ^9^ /L), median(IQR)	4.98(4.65,5.31)	4.85(4.62,5,01)	4.62(4.34,5.15)	0.397	0.225	0.399
RDW (%),median (IQR)	12.69(0.94)	12.33(11.56,12.80)	12.25(11.73,12.70)	0.244	0.253	0.899
PLT(10 ^9^ /L), mean (SD)	206.64(69.78)	242(82.85)	246(52.66)	0.494	0.436	0.714
PDW, median(IQR)	11.65(10.60,14.25)	15.05(11.78,16.08)	13.10(11.85,15.00)	**0.023**	0.126	0.474
MPV, median(IQR)	10.45(9.33,11.83)	10.30(9.53,11.70)	10.10(9.70,10.75)	0.774	0.662	0.483
CRP (mg/L), median(IQR)	6.34(1.48,7.62)	4.29(1.28,7.19)	4.20(1.55,6.33)	0.496	0.497	0.861
FIB(g/L), median(IQR)	2.68(2.22,3.08)	2.78(2.18,2.98)	2.63(2.20,2.74)	0.774	0.771	0.545
UA(µmol/L), median(IQR)	333(261,373)	347(286,480)	307(263,383)	0.238	0.865	0.186
TG(mmol/L), median(IQR)	1.31(0.71,2.40)	1.16(0.99,1.91)	1.02(0.72,1.79)	0.342	0.645	0.324
TC(mmol/L), mean (SD)	4.42(0.78)	4.41(0.94)	4.58(1.19)	0.881	0.376	0.417
LDL(mmol/L), mean (SD)	2.68(0.62)	2.62(0.89)	2.69(0.87)	0.358	0.428	0.611
HDL(mmol/L), mean (SD)	1.24(0.31)	1.26(0.33)	1.37(0.32)	0.927	0.895	0.399
CGRP(pg/mL), median(IQR)	78(73,81)	83(79,87)	91(87,92)	**0.006**	**0.001**	**0.003**
HIT-6 ,mean (SD)	65.57(10.99)	67.88(7.41)	62.00(10.00)	0.229	0.877	0.081

*P*^1^: Grade 1 vs. Grade 2; *P*^2^ : Grade 1 vs. Grade 3; *P*^3^ : Grade 2 vs. Grade 3. The differences were considered significant if *P* value < 0.05

### Correlation analysis between CGRP and HIT-6 score, PFO diameter

**Fig. 3:**
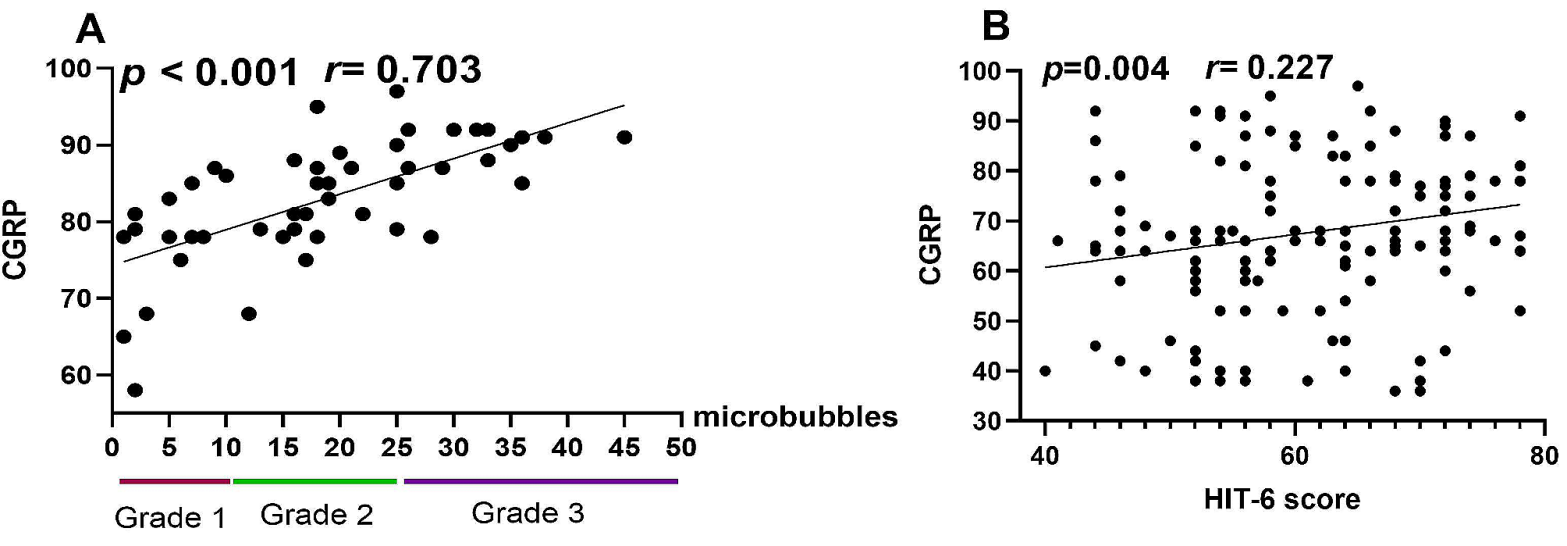
Analysis of correlation between serum CGRP value classification of RLS and HIT-6 score

There was a positive correlation between CGRP and RLS grade (Spearmen rho = 0.703, *P* < 0.001) (Fig. 3 A). Furthermore, we found that there was a positive correlation between CGRP and HIT-6 score in patients with MA with PFO (Spearmen rho = 0.227; *P* = 0.016), while there was no correlation between CGRP and PFO diameter (*P* = 0.083). (Fig. 3 B).

### ROC analysis of CGRP value predicting PFO in MA patients

**Fig. 4:**
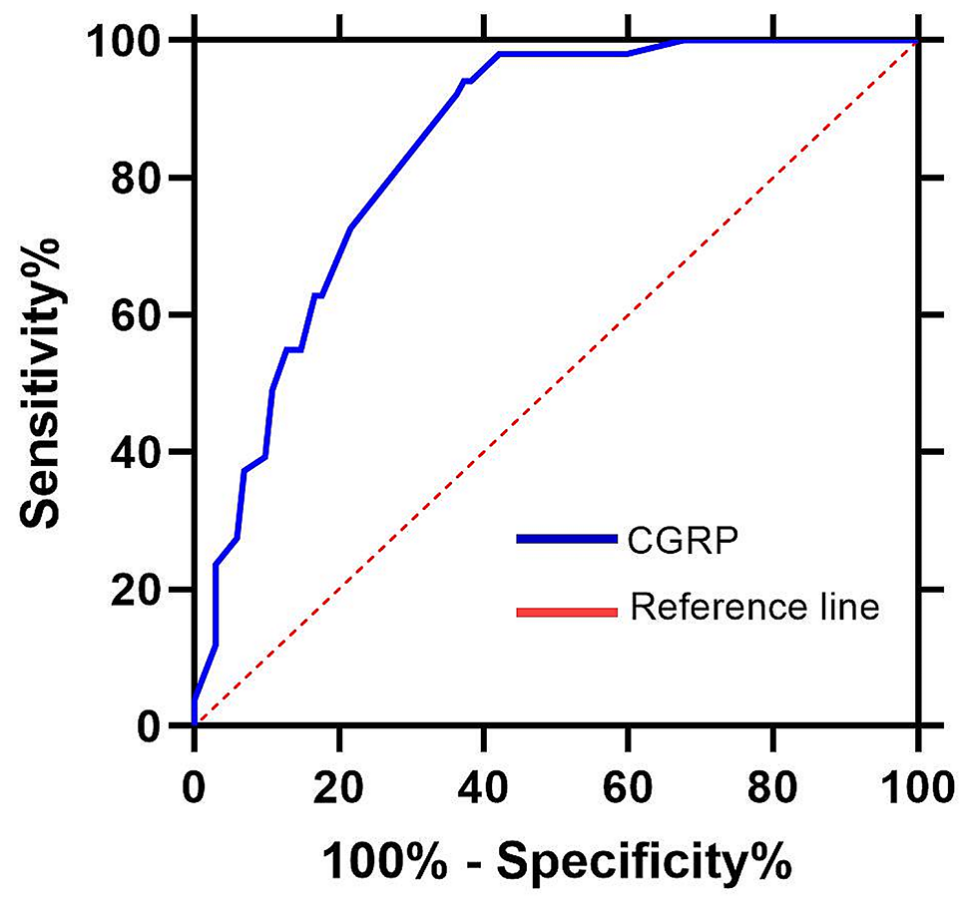
The Relationship between CGRP and MA with PFO

According to the ROC curve analysis, the optimal cut-off value for diagnosing MA with PFO was 79 pg/mL, and the area under the curve (AUC) for predicting RD was 0.845, with 72.55% sensitivity and 78.43% specificity (Fig. 4).

## Discussion

Our results showed that the CGRP in MA with PFO group was significantly higher than that in MA group, and increased CGRP level was associated with elevated RLS grade and HIT-6 score. Furthermore, CGRP value was independently associated with the risk of MA with PFO. Moreover, the cutoff of CGRP in ROC analysis was 79, with the sensitivity 72.55%, and the specificity 78.43%. As far as we know, this study is the first one to explore the correlation between CGRP and MA with PFO, and to use CGRP as the biomarker to evaluate whether MA patients are accompanied by PFO.

### Migraine and PFO

With the development of clinical medicine, accumulating studies have confirmed that PFO is related to MA [5]. It was found that PFO is more common in MA patients with aura [17]. Consistently, aura migraine accounted for 41% of patients with MA with PFO in our study [18]. It is unclear why PFO is associated with migraine. A hypothesis is that many vasoactive substances are usually excreted or metabolized through pulmonary circulation, but venous blood can be shunted directly into arterial blood through the PFO channel without circulation in the lungs. Chemicals and hormones, such as serotonin or CGRP, could bypass the pulmonary circulation, cross the blood-brain barrier directly and stimulate the trigeminal nerve, causing migraines [19, 20].In addition, tiny emboli in the venous circulation can pass and directly enter the arterial system through PFO, triggering low perfusion or cortical spread inhibition, and leading to migraine attacks. Further, Cao et al. provides neuroimaging evidence and new insights into the correlation between PFO and migraine. They found that in migraine patients with PFO, the presence of PFO may affect the structure of the cerebral cortex and the integrity of white matter, which is mainly locked in subcortical, deep white matter, and posterior circulation, and may lead to changes in brain function, such as cerebellum and colliculus, which are involved in the processing and transmission of pain [21].

### CGRP and MA with PFO

The important role of CGRP in the pathophysiology of MA has been proven in many studies. First, CGRP exists in the peripheral sensory nerves of the meninges, and the neurons originating from the meningeal sensory pathway are related to migraine [22]. Secondly, CGRP is associated with pain transmission and inflammation promotion, and exogenous CGRP administration leads to migraine-like headache symptoms in patients with migraine [23]. Moreover, all anti-CGRP agents were more effective than placebo in migraine prevention [24]. Third, CGRP regulates the activity of neurons, glial cells and immune cells in the trigeminal nervous system to promote peripheral and central sensitization and persistent hypervigilance associated with migraine phenotypes [25].

Serum CGRP is a vasoactive polypeptide with a strong expansion effect. Several studies found that serum CGRP levels increased during migraine attacks [26, 27]. Our study found that CGRP level of the MA with PFO group was significantly higher than those without PFO, indicating that less CGRP in MA with PFO patients was metabolized in the pulmonary circulation. In addition, the HIT-6 score of MA with PFO group was significantly higher than that of MA, indicating that patients with MA with PFO suffered more severe headaches.

We further found that higher concentration of CGRP was associated with increased RLS grade. These results suggest that the more RLS shunt flow, the more CGRP bypassing the pulmonary circulation and directly crossing the blood-brain barrier to cause severer migraine attacks in MA patients with PFO.

Some studies have found that RLS grades are related to the severity of migraine [28], but our study did not find a correlation between RLS and HIT-6 scores. The possible reason may be that the sample size of our study is small.

### FIB, MPV and MA with PFO

Moreover, our study found that patients in MA with PFO group had higher levels of MPV and CRP than those in MA group, which was consistent with previous studies. Previos study found that increased level of platelet activation and enhanced release response was common in the attack of MA [29], and the blood MPV in MA patients was significantly higher than that in healthy controls [30].

In addition, our study found that the FIB of MA with PFO group was higher than that of MA group. According to literature, MPV is closely related to platelet activity and participates in inflammation and thrombosis [31], and FIB is associated with thrombus. Our study indicates that MA patients with PFO had increased degree of platelet activation, and slower velocity of venous blood flow, therefore, platelets were more likely to aggregate in the vein to form microemboli, resulting in increased FIB reactivity [32]. This is also consistent with the hypothesis of MA caused by PFO, because RLS leads to decreased blood oxygen saturation and hypoxia, thus increasing the expression of plasminogen activator, such as FIB, which increases the possibility of microembolism, leads to cortical spread inhibition, and leads to MA attack [33].

## Limitation

Our study has some certain limitations. Firstly,, this study is a retrospective study, which can not draw a conclusion on causality, and the popularization of the conclusion still needs to be cautious. Secondly, the short time of this study, strict inclusion of subjects, small sample size and only collecting CGRP during MA attack may have a certain deviation to the results. Thirdly, the onset of MA is affected by many factors, which were not included in this test. Therefore, multicenter experiments with expanded sample, rigorous inclusion and more risk factors are required in the future.

## Conclusions

In summary, this study illustrates that the serum of CGRP in MA patients with PFO is significantly higher than that in MA patients. Furthermore, CGRP is positively associated with RLS grade and HIT-6 score. Serum CGRP has diagnostic predictive value for MA with PFO, which is worthy of further study.Higher serum CGRP level has certain clinical value in predicting PFO in MA patients.

## Data Availability

The data that support the findings of this study are available on request from the corresponding author.

## Abbreviations

CGRP: calcitonin gene-related peptide
MA: migraine
PFO: patent foramen ovale
ROC: receiver operating characteristic
AUC: area under the curve
c-TCD: contrast transcranial Doppler
TTE: transthoracic echocardiography
TEE: transesophageal echocardiography
RLS: right-to-left shunt
HIT-6: headache impact test-6
CRP: C-reactive protein ()
UA: uric acid
TC: total cholesterol
TG: triglycerides
HDL: high-density lipoprotein cholesterol
LDL: low-density lipoprotein cholesterol
FIB: fibrinogen
